# Hematologic biomarkers of aging (HemeAge) and cardiovascular risk: a machine learning analysis in two cohorts

**DOI:** 10.1016/j.ajpc.2026.101460

**Published:** 2026-02-01

**Authors:** Adi Siddharth, David Zidar, Budhaditya Bose, Rakesh Gullapelli, Juan C Nicholas, Khurram Nasir, Sadeer Al-Kindi

**Affiliations:** a Center for Cardiovascular Computational and Precision Health, Department of Cardiology, Houston Methodist, Houston, TX, USA; b Department of Cardiovascular Medicine, Cleveland Clinic Foundation, Cleveland, OH, USA

**Keywords:** Hemeage, Cardiovascular disease, Machine learning, Complete blood count, Risk Stratification

## Abstract

**Background:**

Chronological age inadequately reflects aging variability and cardiovascular risk. Biological age derived from routine complete blood count (CBC) parameters may provide a more actionable marker.

**Objective:**

To develop a machine learning model of biological age using CBC data (HemeAge) and evaluate associations with mortality and major adverse cardiovascular events (MACE) in two large cohorts.

**Methods:**

An XGBoost model was trained on 53,355 NHANES participants (1999–2010) to predict chronological age from CBC parameters. The model was applied to 109,844 Houston Methodist CVD Registry patients, generating "delta age" (predicted minus chronological age). Patients were classified as Resilient (delta < –10), Proportionate (–10 ≤ delta ≤ 10), or Accelerated (delta > 10). Cox models assessed mortality and MACE risk, adjusting for demographics and clinical factors.

**Results:**

Red cell distribution width, mean cell volume, and neutrophil count were key age predictors. Accelerated aging associated with increased mortality risk (HR 3.05, 95% CI 2.41–3.85) and MACE (HR 1.37, 95% CI 1.24–1.51) versus proportionate aging. Resilient aging conferred reduced risk for mortality (HR 0.59, 95% CI 0.52–0.68) and MACE (HR 0.76, 95% CI 0.72–0.81). Associations were strongest in midlife (ages 40–80) and for death and heart failure outcomes and persisted across age-stratified and continuous models.

**Conclusions:**

HemeAge independently predicts mortality and cardiovascular risk beyond chronological age. These accessible hematologic markers may enhance risk stratification and inform targeted prevention strategies.

## Highlights

• Machine-learning model developed to estimate biological age from CBC parameters.
• Model trained in 53,355 NHANES participants and applied to 109,844 hospital patients.
• CBC-derived HemeAge phenotypes is associated mortality independent of demographics and comorbidities.
• Accelerated aging showed higher mortality and MACE risk; resilient aging showed lower risk.
• CBC-based biological age offers an accessible tool for cardiovascular risk stratification.

## 1 Introduction

Chronological age is increasingly recognized as an imperfect marker of health, as individuals of the same age often show wide variation in ageing outcomes. This has shifted attention to biological age, which reflects the cumulative effects of genetics, lifestyle, and disease on physiological and functional status, is considered modifiable, and offers insight into individual ageing trajectories through measures such as age acceleration [1]. Cardiovascular disease (CVD) remains a leading cause of death worldwide; despite advances in prevention, an ageing population and rising early-onset cases sustain its burden [2]. The progressive deterioration associated with ageing is a fundamental risk factor for chronic diseases, including CVD, [3] making biologic age assessment via physiological, metabolic, and functional markers an important tool for understanding links between ageing and cardiovascular health [1].

A significant aspect of biological aging involves systemic alterations that are often evident in routine blood tests. Immunosenescence, an age-related decline in immune function, is characterized by impaired immune responses and increased susceptibility to both infectious and chronic diseases. A key downstream effect of immunosenescence is “inflammageing,” a persistent, low-grade inflammatory state that serves as a fundamental pathophysiological mechanism and a major risk factor for many aging-related conditions, including CVD [4].

Routine complete blood count (CBC) parameters have emerged as accessible biomarkers reflecting these processes. Several CBC measures have shown dynamic changes with aging, particularly changes in white blood cell composition. For example, several studies have shown that absolute lymphocyte count (ALC) declines with age [5,6]. These changes are not universal and heterogeneity may reflect different aging trajectories. These hematologic alterations appear to have prognostic implications across various health conditions. For example, red cell distribution width, [[7], [8], [9], [10]] which measures red cell size variability, and ALC [11,12] are strong predictors of mortality, CVD, infections, and cancers. Other markers, such as neutrophil counts, [13] platelet counts, [14] mean platelet volume, [15] eosinophil count, [16] monocyte counts, [17] and others have shown relationships with various health outcomes. Some combinations of these markers, such as neutrophil to lymphocyte ratio, [18] platelet to lymphocyte ratio, [19] RDW to platelet ratio, [20] and others have also been linked with various health outcomes.

To our knowledge, no prior study has incorporated all these measures to evaluate accelerated and resilient aging trajectories. Therefore, we aimed to develop and validate a HemeAge framework to understand heterogeneity in hematologic changes with age, and create phenotypes that may have prognostic implications across health outcomes.

## 2 Methods

### 2.1 Development of the CBC-Age model in NHANES

We used data from the National Health and Nutrition Examination Survey (NHANES) from 1999 to 2010 to train a supervised machine learning model for estimating biological age (model predictions will henceforth be labelled as HemeAge). Complete blood count (CBC) variables and demographic information were extracted from NHANES participants, resulting in a training cohort of 53,355 individuals. An XGBoost regression model was trained using only CBC parameters as predictors, with chronological age as the target variable. We selected a panel of hematologic biomarkers, including the following variables: White Blood Cell Count, Lymphocyte Percent, Lymphocyte Number, Monocyte Percent, Monocyte Number, Neutrophil Percent, Neutrophil Number, Eosinophil Percent, Eosinophil Number, Basophil Percent, Basophil Number, Red Blood Cell Count, Hemoglobin, Hematocrit, Mean Cell Volume, Mean Cell Hemoglobin, Mean Cell Hemoglobin Concentration, Red Cell Distribution Width, Platelet Count.

### 2.2 Application to the houston methodist cohort

The Houston Methodist (HM) Cardiovascular Disease (CVD) Registry [21] is an automated, electronic medical record–based learning health system registry that captures longitudinal outpatient data for adult patients receiving care across the HM health system, including individuals with established atherosclerotic cardiovascular disease (ASCVD) as well as those at risk for ASCVD. The registry integrates demographic data, diagnoses, laboratory results, medications, and comorbidities using validated ICD-10 codes and standardized extraction pipelines, as previously described. We extracted data from the HM CVD Registry using Structured Query Language (SQL). The registry includes all adult patients (≥18 years) with at least one outpatient visit to HM physicians between June 2016 and August 2023. For the present study, inclusion did not require a prior diagnosis of ASCVD.

Records were included if the lab values were numeric, non-missing, and accompanied by valid units of measurement. For each patient, we retained only the earliest outpatient CBC encounter to ensure a consistent baseline across the cohort. From the refined dataset, The CBC data was then merged with demographic information including age, sex, race, ethnicity. Race and ethnicity were harmonized into Hispanic, Non-Hispanic Asian, Non-Hispanic Black, Non-Hispanic Other, Non-Hispanic White. We also incorporated geographic vulnerability indicators by linking each patient’s census tract to the CDC Social Vulnerability Index (SVI), [22] which provides percentile ranks across four thematic domains and an overall summary score. Preexisting comorbidities were identified by mapping diagnostic codes from medical history, problem lists, claims, and other clinical documentation to standard comorbidity categories based on the Charlson Comorbidity Index. Diagnoses were included only if they preceded or coincided with the CBC specimen date, and the resulting comorbidity indicators were incorporated into the final dataset The final analytic dataset included each patient’s earliest CBC results along with demographic variables, geographic identifiers, comorbidity indicators, and SVI measures.

To validate the HemeAge model, we applied the trained NHANES XGBoost model to a separate cohort of patients from the HM CVD Registry. This cohort included individuals with available CBC measurements that matched the variables used in NHANES. The model generated a predicted age for each patient, referred to as “HemeAge”. We then calculated a “delta” value as the difference between predicted biological age and chronological age. Based on this continuous delta, patients were categorized into three HemeAge groups: (1) Proportionate: delta between −10 and +10 years; (2) Resilient: delta <−10 years; (3) Accelerated: delta >+10 years. The delta and age groups were used as the primary exposure variable in downstream survival analyses

We used Cox proportional hazards regression models to examine the association between HemeAge categories (delta and age groups) and time-to-event outcomes, adjusting for demographic, clinical, and social factors. Observations with missing data in any predictors or outcome variables were excluded. The age groups variable was modeled as an ordinal variable and one-hot encoded, with the “Proportionate” group designated as the reference. Additional covariates included chronological age, sex, race/ethnicity, Charlson comorbidity indicators, and Social Vulnerability Index (SVI) scores, all of which were encoded using dummy variables where applicable. Predictors with near-zero variance were removed to ensure model stability. Models were estimated using partial likelihood, and results are reported as hazard ratios with 95 % confidence intervals. Cox proportional hazards models were used to evaluate the association between HemeAge categories and clinical outcomes. The primary outcomes of interest were all-cause mortality and major adverse cardiovascular events (MACE). MACE was defined as a composite of myocardial infarction, stroke, heart failure, and all-cause mortality.

Covariates in the adjusted models included chronological age, sex, race/ethnicity, comorbidity indicators derived from the Charlson Comorbidity Index, and Social Vulnerability Index scores linked via census tract. Models were fit using partial likelihood estimation, and results were reported as hazard ratios with 95 % confidence intervals. Three cox models were constructed: Model 1 (M1) adjusts for chronological age, sex, and race and ethnicity; Model 2 (M2) adds comorbidities (Charlson comorbidity index); Model 3 (M3) further includes social vulnerability index.

### 2.3 Subgroup and secondary analyses

We conducted stratified analyses to assess whether the relationship between HemeAge and outcomes varied across age groups. Patients were categorized into four age strata: <40, 40–60, 60–80, and >80 years. Within each stratum, separate Cox models were constructed, adjusting for chronological age, sex, and race/ethnicity.

To better understand the contribution of each component within the MACE composite, we conducted separate Cox models for each individual event: stroke, myocardial infarction, heart failure, and all-cause mortality. These models were adjusted for chronological age, sex, race/ethnicity, comorbidities, and SVI. Data analysis was performed using Python 3 and querying was performed using SQL. The study was approved by the Houston Methodist Institutional Review Board.

Portions of code generation, data visualization, and manuscript language editing were assisted by using LLMs, with all analyses, interpretations, and final text verified by the authors.

## 3 Results

### 3.1 NHANES

The NHANES cohort used for the CBC analysis included a diverse, nationally representative sample with near-equal gender distribution across all racial and ethnic subgroups. Among participants, non-Hispanic White individuals comprised the largest subgroup, representing 43.3% of the total population (11,365 males and 11,745 females). This group also had the highest median age, with males having a median age of 52 years and females a median age of 50 years.

Non-Hispanic Black participants accounted for 20.9% of the cohort (5,356 males and 5,789 females), with a median age of 46 years for males and 45 years for females. Mexican American individuals made up 18.5% of the cohort (4,740 males and 5,136 females) and were generally younger, with a median age of 42 years in males and 41 years in females. Other Hispanic participants represented 8.1% of the cohort (1,915 males and 2,433 females), with median ages of 47 years for males and 46 years for females. Individuals identifying as other race, including multiracial, comprised 9.1% of the cohort (2,387 males and 2,489 females), with a median age of 43 years for both males and females.

The relationship between predicted biological age and true chronological age is illustrated in Figure S1. The prediction model, trained on NHANES CBC data, showed moderate correlation with actual age (R² = 0.20), indicating notable variability in age predictions. The overall prediction root mean square error (RMSE) was 15.79 years, highlighting heterogeneity in HemeAge relative to chronological age across the population.

**Fig. 1:**
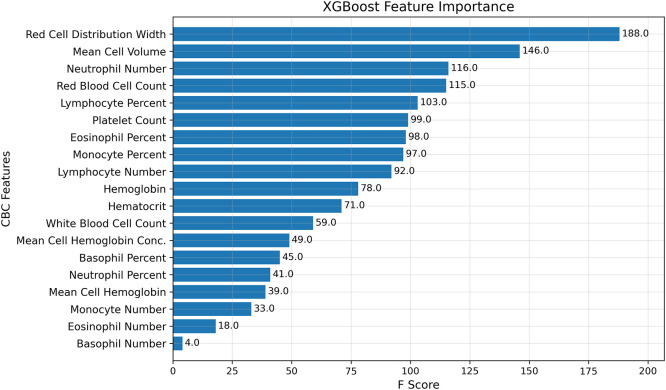
Feature importance scores from the XGBoost model trained on NHANES.

Feature importance analysis from the XGBoost model trained on NHANES CBC data identified several hematologic parameters as key predictors of biological age (Fig. 1). Red cell distribution width (RDW) was the most influential feature, followed by mean cell volume (MCV), absolute neutrophil count, and red blood cell count, with additional contributions from other leukocyte subtypes and standard CBC measures.

Partial dependence plots for the four most influential features (Figure S2) showed that higher RDW and MCV were associated with increasing predicted biological age, whereas higher red blood cell counts were associated with lower predicted age. Neutrophil number exhibited a non-linear pattern, with predicted age plateauing across mid-range values and decreasing at the extremes.

**Fig. 2:**
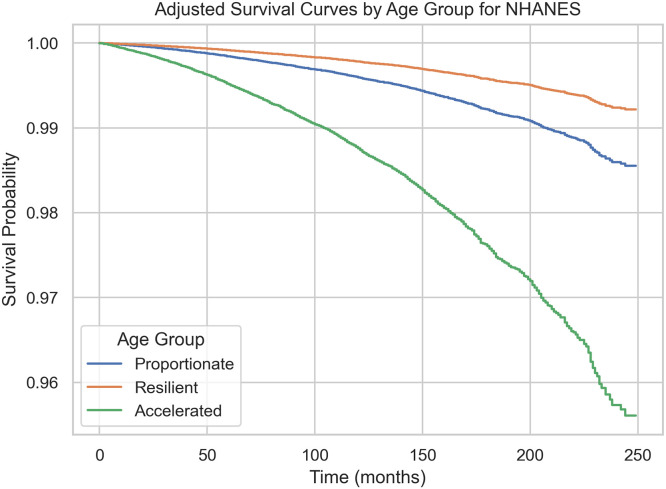
Adjusted survival curves for cardiovascular-related mortality in the NHANES cohort, stratified by CBC delta age group.

Fig. 2 shows cause-specific survival for cardiovascular-related mortality in the NHANES cohort, adjusted for chronological age, sex, and race/ethnicity. The accelerated HemeAge group experienced the steepest decline in survival, whereas the resilient group had the most favorable trajectory, with the proportionate group intermediate.

### 3.2 Houston methodist

Table 1 summarizes baseline demographic and clinical characteristics of 109,844 Houston Methodist patients with available complete blood count (CBC) with differential data, stratified by HemeAge phenotype: proportionate (*n* = 47,782), resilient (*n* = 30,952), and accelerated HemeAge (*n* = 31,110). Overall, the cohort was predominantly female, with resilient (62.3 %) and accelerated (63.5 %) HemeAge groups having slightly higher proportions compared to the proportionate HemeAge group (57.6 %). Non-Hispanic White individuals were the largest subgroup across all phenotypes, particularly within the resilient group (63.3 %), while Hispanic individuals had notably higher representation in the accelerated group (21.7 %).

**Table 1:** Baseline characteristics for the Houston Methodist patient population.

Characteristic	Overall	Proportionate	Resilient	Accelerated
Sample Size, n	109,844	47,782	30,952	31,110
Gender				
Female	66,584 (60.6 %)	27,541 (57.6 %)	19,297 (62.3 %)	19,746 (63.5 %)
Male	43,260 (39.4 %)	20,241 (42.4 %)	11,655 (37.7 %)	11,364 (36.5 %)
Race Ethnicity				
Hispanic	18,768 (17.1 %)	8283 (17.3 %)	3719 (12.0 %)	6766 (21.7 %)
Non-Hispanic Asian	7283 (6.6 %)	3396 (7.1 %)	1601 (5.2 %)	2286 (7.3 %)
Non-Hispanic Black	14,817 (13.5 %)	6359 (13.3 %)	4528 (14.6 %)	3930 (12.6 %)
Non-Hispanic Other	7377 (6.7 %)	3274 (6.9 %)	1510 (4.9 %)	2593 (8.3 %)
Non-Hispanic White	61,599 (56.1 %)	26,470 (55.4 %)	19,594 (63.3 %)	15,535 (49.9 %)
True Age				
Median (IQR)	48.00 (34.00–63.00)	48.00 (40.00–56.00)	67.00 (61.00–74.00)	29.00 (23.00–35.00)
Predicted Age				
Median (IQR)	47.66 (42.14–54.00)	47.46 (41.41–54.65)	47.86 (42.61–53.56)	47.72 (42.71–53.56)
Delta				
Median (IQR)	0.19 (−11.61–11.59)	0.15 (−4.82–4.99)	−17.86 (−23.74–−13.65)	17.52 (13.51–22.76)
SVI (Social Vulnerability Index)				
Median (IQR)	0.40 (0.17–0.66)	0.39 (0.17–0.65)	0.42 (0.17–0.67)	0.40 (0.18–0.66)

**Fig. 3:**
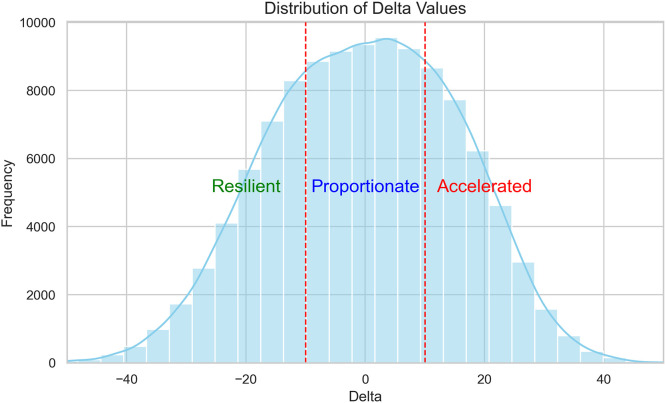
Distribution of delta age values for Houston Methodist population.

HemeAge were similar across resilient, proportionate, and accelerated groups (∼48 years) whereas resilient patients were more likely to be chronologically older (mean: 67.89 ± 9.91 years), compared to proportionate patients (mean: 48.35 ± 11.47 years), and accelerated patients (mean: 29.78 ± 8.66 years). The overall distribution of delta age values approximated a normal distribution centered around zero (Fig. 3). The mean difference between chronologic age and HemeAge was −19.50 ± 7.39 years for the resilient group, 0.09 ± 5.72 years for the proportionate group, and 18.81 ± 6.62 years for the accelerated group. Social Vulnerability Index (SVI) scores were similar across groups.

**Fig. 4:**
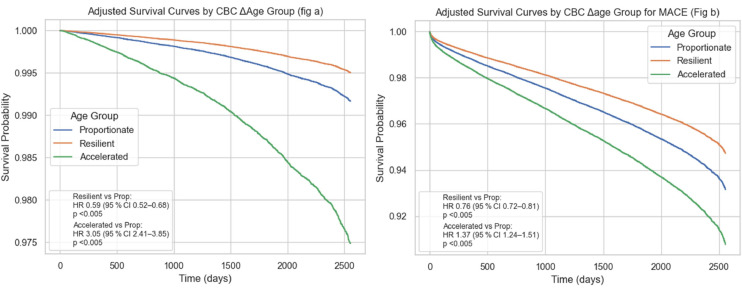
Adjusted survival curves by HemeAge group. (a) Adjusted survival for all-cause mortality by biological aging phenotype. (b) Adjusted survival for major adverse cardiovascular events (MACE) by biological aging phenotype.

For all-cause mortality (Fig. 4a), survival probability declined most steeply in the accelerated aging group, with an adjusted hazard ratio (HR) of 3.05 (95 % CI 2.41–3.85; p < 0.005) compared with the proportionate reference group. The resilient group demonstrated a more favorable survival trajectory, with a significantly reduced mortality risk (HR 0.59, 95 % CI 0.52–0.68; p < 0.005).

A similar pattern was observed for major adverse cardiovascular events (MACE) (Fig. 4b). The accelerated group again exhibited the highest event rates over time, with an adjusted HR of 1.37 (95 % CI 1.24–1.51; p < 0.005) relative to the proportionate group. In contrast, the resilient group showed significantly lower risk (HR 0.76, 95 % CI 0.72–0.81; p < 0.005). Overall, these curves demonstrate that accelerated HemeAge is associated with poorer survival, while resilient HemeAge confers a protective effect, independent of chronological age and other covariates.

Table 2 present hazard ratios for mortality and MACE across chronological age strata, comparing delta age groups to the proportionate reference group. For mortality, the accelerated group was consistently at higher risk, with hazard ratios of 4.91 and 4.94 in the 40–60 and 60–80 age ranges, respectively (both p < 0.005). Elevated risk was also observed under age 40 (HR 2.87, p = 0.024), though with wider confidence intervals. In contrast, the resilient group showed significantly lower mortality risk in the 60–80 (HR 0.49, p < 0.005) and >80 (HR 0.56, p = 0.012) age groups. No significant difference was seen in the 40–60 range (HR 0.72, p = 0.112), and no deaths occurred in the resilient group under age 40 or in the accelerated group over 80.

**Table 2:** Age-stratified Cox proportional hazards model results for mortality and major adverse cardiovascular events (MACE) by HemeAge phenotype.

Age (Years)	Accelerated			Resilient		
	HR	95 % CI	P Value	HR	95 % CI	P Value
Mortality						
<40	2.87	(1.15, 7.2)	0.024			
40–60	4.91	(3.53, 6.82)	<0.005	0.72	(0.48, 1.08)	0.112
60–80	4.94	(2.98, 8.21)	<0.005	0.49	(0.42, 0.57)	<0.005
>80				0.56	(0.36, 0.88)	0.012
MACE						
<40	1.19	(0.94, 1.5)	0.151			
40–60	2.4	(2.09, 2.75)	<0.005	0.8	(0.7, 0.91)	<0.005
60–80	2.11	(1.53, 2.9)	<0.005	0.65	(0.61, 0.69)	<0.005
>80				0.53	(0.43, 0.67)	<0.005

Hazard ratios (HR) and 95 % confidence intervals (CI) are shown for the accelerated and resilient aging groups compared with the proportionate aging reference group, adjusted for chronological age, sex, and race/ethnicity. Results are stratified by chronological age categories (<40, 40–60, 60–80, and >80 years). A blank cell indicates no events in that group. Significant associations (p < 0.005 unless otherwise stated) are highlighted in the text.

For MACE (Table 2b), a similar pattern was observed. The accelerated group again demonstrated significantly increased risk in the 40–60 (HR 2.40) and 60–80 (HR 2.11) age groups (both p < 0.005), while the association was not significant under age 40 (HR 1.19, p = 0.151). The resilient group showed significantly lower risk of MACE in the 40–60 (HR 0.80), 60–80 (HR 0.65), and >80 (HR 0.53) strata (all p < 0.005). As with mortality, no events were observed in the resilient group under age 40 or in the accelerated group over 80 due to small sample size. Corresponding age-stratified models additionally adjusted for Charlson Comorbidity Index are provided in the Supplement (Table S6).

Table S1 shows age-stratified incidence rates of MACE and mortality by HemeAge phenotype. Event rates were very low under age 40, with no deaths in the resilient group. In the 40–60 range, the accelerated group had the highest rates for both MACE (2.39 per 100 PY) and mortality (0.47), exceeding those in the proportionate and resilient groups. In the 60–80 range, mortality was again highest in the accelerated group (3.74 per 100 PY), while MACE rates were elevated across all groups. Among those over 80, no accelerated patients were present; the resilient group showed lower mortality (3.32) than the proportionate group (5.07).

Figure S3a and S3b show survival probabilities by CBC Δ-age group in younger and older participants, respectively. In patients under 65 years (Figure S3a), the Accelerated group demonstrated substantially lower survival, whereas the Resilient group had the most favorable trajectory over time. Similarly, among patients aged 65 years and older (Figure S3b), Accelerated aging was again associated with reduced survival, while the Resilient group maintained higher survival probabilities throughout follow-up.

Table S2 summarizes the performance of individual Cox models across MACE outcomes, with C-indices ranging from 0.81 to 0.86. The Resilient group showed significantly lower risk of heart failure (HR 0.68), myocardial infarction (HR 0.76), and death (HR 0.59), all with p-values <0.005; no significant difference was observed for stroke (HR 0.95, *p* = 0.28). The Accelerated group had significantly higher risk for heart failure (HR 1.45, *p* < 0.005), myocardial infarction (HR 1.23, *p* = 0.02), and death (HR 3.05, *p* < 0.005), while the association with stroke was not statistically significant (HR 1.17, *p* = 0.07). Figures S4a–c illustrates survival curves for individual cardiovascular outcomes by delta age group.

In all three models, the Accelerated group consistently had the lowest survival probability across the follow-up period, while the Resilient group showed the most favorable survival, followed by the Proportionate group. For stroke (Figure S4a), the separation between groups was modest, with survival curves remaining relatively close together and no statistically significant differences.

For myocardial infarction (Figure S4b), differences were more evident, with the Resilient group showing reduced risk and the Accelerated group showing increased risk compared with the Proportionate group.

For heart failure (Figure S4c), the separation between groups was most pronounced, with clear divergence early in follow-up and the Accelerated group demonstrating the steepest decline in survival. These results align with the pattern seen in the composite MACE analysis. Tables S4a and S4b show that model discrimination improved from a C-index of 0.84, when only age, sex, and race/ethnicity were included, to 0.86 after adding the Charlson Comorbidity Index; adding the Social Vulnerability Index did not raise the C-index further. With HemeAge coded as a categorical phenotype (Table S4), the Resilient group consistently exhibited lower mortality risk (fully adjusted HR 0.59, 95 % CI 0.52–0.68; p < 0.005), whereas the Accelerated group experienced roughly a three-fold increase in risk (HR 3.05, 2.41–3.85). When biological ageing was modeled as a continuous delta age (Table S4b), each one-unit increase in delta age independently raised mortality hazard by about 4 % (HR 1.04, 1.03–1.05). Male sex and greater comorbidity remained strong predictors across all models, non-Hispanic Asian ethnicity was protective, and SVI itself showed no independent association with mortality once other factors were accounted for.

Table S5 displays results from Cox models evaluating the association between delta age and MACE using two approaches: age group classification and continuous delta. In all models, delta age was significantly associated with MACE, independent of chronological age and other covariates. In the age group models (Tables S5), the Accelerated group had significantly increased risk (HR 1.63 in M1 to 1.36 in M3), while the Resilient group consistently showed reduced risk (HRs 0.68 to 0.77). In the continuous delta models (Tables S5), each unit increase in delta age was associated with a modest but statistically significant increase in MACE risk (HRs 1.029 to 1.019). Adding comorbidities improved model performance (C-index rose from 0.798 to 0.800 to 0.838), with minimal further change after adjusting for social vulnerability

## 4 Discussion

Across both the Houston Methodist and NHANES cohorts, HemeAge phenotypes derived from CBC data were strongly associated with clinical outcomes. Patients with accelerated HemeAge consistently demonstrated worse cardiovascular and survival outcomes than those with proportionate or resilient HemeAge. In the Houston Methodist cohort, accelerated HemeAge was associated with higher risk of all-cause mortality and MACE after adjustment for chronological age, sex, race/ethnicity, comorbidities, and social vulnerability. These associations persisted across most age strata, with the greatest excess risk observed in midlife (40–60 and 60–80 years). In contrast, resilient HemeAge was associated with lower hazards and more favorable survival trajectories, particularly in older individuals despite their chronologically older profiles.

The accelerated HemeAge phenotype was more frequently observed among chronologically younger individuals, reflecting discordance between biological and chronological age and raising concern for lower event rates in this group. Age-stratified analyses showed sparse event accrual in certain phenotype–age strata, particularly among resilient individuals younger than 40 years and accelerated individuals older than 80 years, resulting in unstable estimates. In contrast, the 40–60 and 60–80 year strata demonstrated substantial representation of all phenotypes and the most consistent bidirectional separation of risk. All models adjusted for chronological age, mitigating confounding by age distribution and supporting the robustness of HemeAge associations where sufficient events were observed.

These findings were replicated in the NHANES cohort despite its different demographic structure. Accelerated HemeAge was again associated with steeper declines in survival free of cardiovascular death, whereas resilient HemeAge was protective. Associations were consistent across both categorical phenotype and continuous delta age models, underscoring HemeAge as an independent marker of cardiovascular risk beyond chronological age and traditional risk factors.

These findings extend preexisting literature on biologic aging and age acceleration/resilience. In our work, we define a predicted biological age from routine CBC markers and calculate a delta to categorize individuals into Proportionate, Resilient, and Accelerated groups. This mirrors prior approaches that used other markers, such as epigenetic markers, proteomics, and other but extends it to a routine, pragmatic, approach. Zhang et al. also describes biological age as distinct from chronological age and use residuals or differences between predicted and actual age to quantify “age acceleration.” [23] These methods align with broader discussions about biological age as a more precise indicator of healthspan, reinforcing that our methodology is in step with emerging standards in ageing research [24].

Interestingly, our unbiased approach has identified RDW as the most important contributor to HemeAge. Prior studies have shown that RDW is linked with various adverse outcomes, including heart failure, stroke, cardiovascular death, [25] COVID19 severity and mortality, and other outcomes. The biologic mechanisms linking RDW with health outcomes remain unclear. Studies have shown that RDW correlates with tumor necrosis levels and other markers of immune activation, [7] programmed cell death 1 (PD-1) expression on CD4 T cells, [26] disrupted iron metabolism, [27] and fibrosis markers [28]. Consistent with these prior associations, secondary analyses of individual cardiovascular outcomes in our cohort demonstrated that accelerated HemeAge, characterized in part by higher RDW, was most strongly associated with myocardial infarction and heart failure, whereas associations with stroke were more modest. We now extend these findings to suggest that RDW varies with age, and that variation contributes to the overall ageing phenotypes observed.

Feature importance and partial dependence analyses from the XGBoost model provide insight into which CBC parameters most strongly contribute to separation between resilient and accelerated HemeAge phenotypes. Red cell distribution width (RDW) emerged as the most influential predictor, followed by mean corpuscular volume (MCV), red blood cell (RBC) count, and absolute neutrophil count. Higher RDW and MCV values were associated with higher predicted biological age, whereas higher RBC counts were associated with lower predicted biological age. Neutrophil count demonstrated a nonlinear relationship, with predicted age increasing across low-to-moderate values and plateauing or declining at higher extremes.

Because HemeAge phenotypes are defined by the difference between predicted biological age and chronological age, these feature-level directions imply that elevated RDW and MCV and lower RBC counts shift individuals toward an accelerated aging phenotype, whereas the opposite patterns are characteristic of resilient aging.

The addition of CBC-derived biological age to existing CV risk models represents an opportunity to enhance precision in clinical risk stratification. Unlike more complex or costly biomarkers (e.g. epigenetic clocks, proteomic clocks), CBC measures are universally available, inexpensive, and routinely collected across healthcare systems. Integrating delta HemeAge into established tools like the PREVENT risk calculator could improve identification of high-risk individuals who might otherwise be underestimated by chronological age alone, particularly among the midlife population (ages 40–60), where our results show the strongest predictive signal. This can present an opportunity for population HemeAging framework, which can provide a window where early detection of accelerated biological aging could prompt precision population preventive therapies. Future studies should explore the additive value of CBC-derived delta age in prospective cohorts and its feasibility as a trigger for guideline-based interventions. Additionally, HemeAge could act as a surrogate in interventional trials, where small studies can be designed to understand the impact on HemeAge and forecast these changes with health outcomes.

Given the simplicity and ubiquity of CBC testing, our approach lends itself to broader applications beyond CV risk prediction. Stratifying HemeAge using routine hematologic parameters could prove useful in oncology, infectious diseases, autoimmune conditions, and frailty-related syndromes, where inflammation, immune competence, and hematopoietic reserve are central to outcomes. Moreover, as global healthcare increasingly focus on healthspan and resilience, delta HemeAge may provide a pragmatic surrogate to monitor population health and disparities over time. This is especially helpful in resource-limited settings. Applying this model across diverse clinical contexts will require recalibration and validation in subgroups with distinct baseline hematologic norms (e.g., by race/ethnicity, sex, geography, or nutritional status) and disease burden.

The findings of this study should be interpreted within the context of its strengths and limitations. Strengths include nationally representative sample for derivation (NHANES), and validation in a large electronic health record system data with clinical outcomes. Limitations include that the model estimates HemeAge from a single baseline CBC, limiting the ability to capture the dynamic and cumulative nature of biological aging over time. Second, the model was trained on NHANES data collected up to 2010, which may not fully reflect evolving epidemiologic trends, such as the stagnation and reversal of cardiovascular mortality declines. Moreover, validation relied on a single-center Houston Methodist cohort, which may not generalize broadly. Lastly, defining MACE as a composite that includes all-cause mortality and categorizing HemeAge using fixed ±10-year thresholds could obscure more specific cardiovascular associations and continuous variation within the biological age spectrum.

## Author agreement

We, the undersigned, certify that this manuscript entitled:

“HemeAge: A Machine Learning Framework for Biological Age Assessment Using Complete Blood Count Parameters” is an original work and is not under consideration for publication elsewhere, nor has it been previously published in whole or in part.

All listed authors meet the criteria for authorship, have reviewed and approved the final version of the manuscript, and agree to its submission to the American Journal of Preventive Cardiology. All authors agree to be accountable for all aspects of the work and to ensure that questions related to the accuracy or integrity of any part of the work are appropriately investigated and resolved.

Any potential conflicts of interest have been disclosed in the manuscript. Ethical approval was obtained as described in the Methods section, and all applicable institutional and regulatory guidelines were followed.

## Data and code availability

Patient-level EHR data from Houston Methodist are not publicly available due to institutional privacy policies and U.S. HIPAA regulations. These data contain protected health information and cannot be shared outside the institution.

Aggregate summary data underlying the main figures and results (counts, event rates, hazard ratios, and 95 % confidence intervals by predefined strata), as well as model specifications, can be made available upon reasonable request to the corresponding authors, subject to Houston Methodist revie.

Publicly available datasets used in this study include the National Health and Nutrition Examination Survey (NHANES) — available at https://www.cdc.gov/nchs/nhanes/index.html and the CDC/ATSDR Social Vulnerability Index (SVI) — available at https://www.atsdr.cdc.gov/place-health/php/svi/index.html.

Custom analysis code operates on restricted institutional EHR data and cannot be released publicly because of privacy and security constraints. To support reproducibility, detailed algorithmic descriptions of data preprocessing, model training, and statistical analyses are provided within the Methods section and Supplementary Information. Executable code cannot be shared or accessed outside Houston Methodist.

## Ethics statement

We utilized an automated, electronic medical record (EMR)-integrated registry previously established at Houston Methodist Hospital (HMH) to identify and longitudinally evaluate patients with established or at-risk atherosclerotic cardiovascular disease (ASCVD). This registry is powered by the unified Epic EMR used throughout the HMH system (eight hospitals and 27 cardiovascular programs). The Houston Methodist Online Research Technology Initiative (MORTI) classified this as a non-interventional, retrospective cohort study. The research was approved under an Institutional Review Board (IRB) under protocol number IRB #: PRO00025790.

## CRediT authorship contribution statement

**Adi Siddharth:** Writing – review & editing, Writing – original draft, Visualization, Software, Methodology, Investigation, Formal analysis, Conceptualization. **David Zidar:** Writing – review & editing. **Budhaditya Bose:** Data curation. **Rakesh Gullapelli:** Data curation. **Juan C Nicholas:** Data curation. **Khurram Nasir:** Writing – review & editing, Supervision. **Sadeer Al-Kindi:** Writing – review & editing, Supervision, Methodology, Investigation, Conceptualization.

## Declaration of competing interest

The authors declare that they have no known competing financial interests or personal relationships that could have appeared to influence the work reported in this paper.

## Appendix Supplementary materials

Image, application 1
