# Supplementary material for: Hematologic biomarkers of aging (HemeAge) and cardiovascular risk: a machine learning analysis in two cohorts
Source: Am J Prev Cardiol. 2026 Feb 1;26:101460. doi: 10.1016/j.ajpc.2026.101460 (PMC13084135; doi:10.1016/j.ajpc.2026.101460)
Supplement: Supplementary file 1 [file mmc1.docx]

**Supplemental Data**


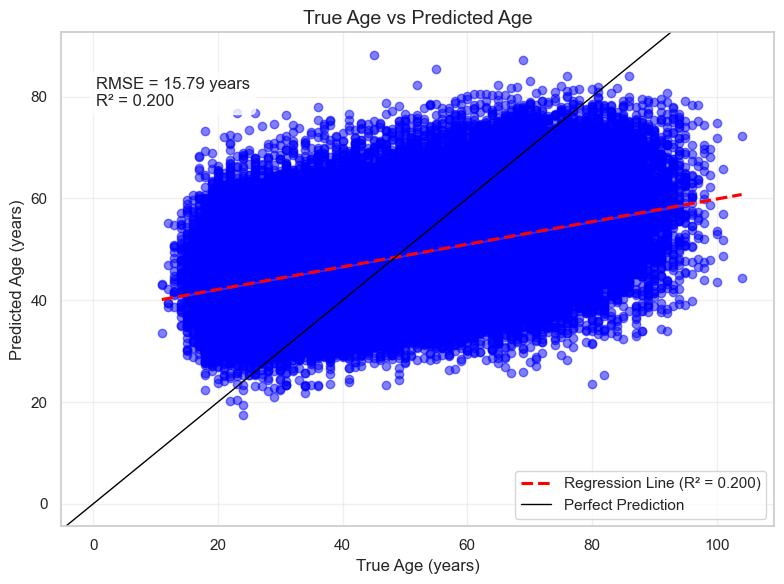


**Figure S1:** Scatter plot of predicted biological age versus true chronological age.


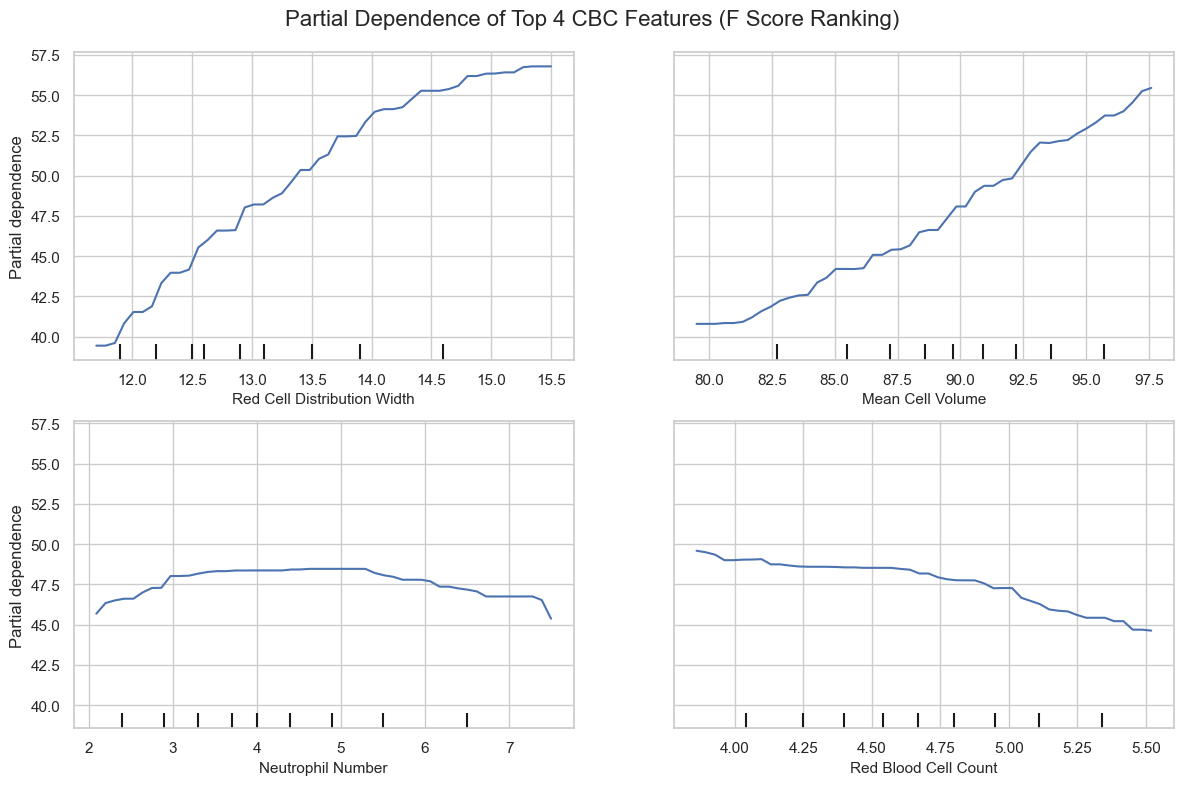


**Figure S2.** Partial dependence plots for the top four CBC features ranked by XGBoost feature importance. Each plot shows the marginal effect of a single feature on predicted biological age while holding all other variables constant. RDW and MCV exhibit positive associations with predicted age, RBC count shows an inverse association, and neutrophil number displays a non‑linear pattern with a plateau across mid‑range values. Tick marks on the x‑axes indicate the distribution of observed feature values in the NHANES dataset.


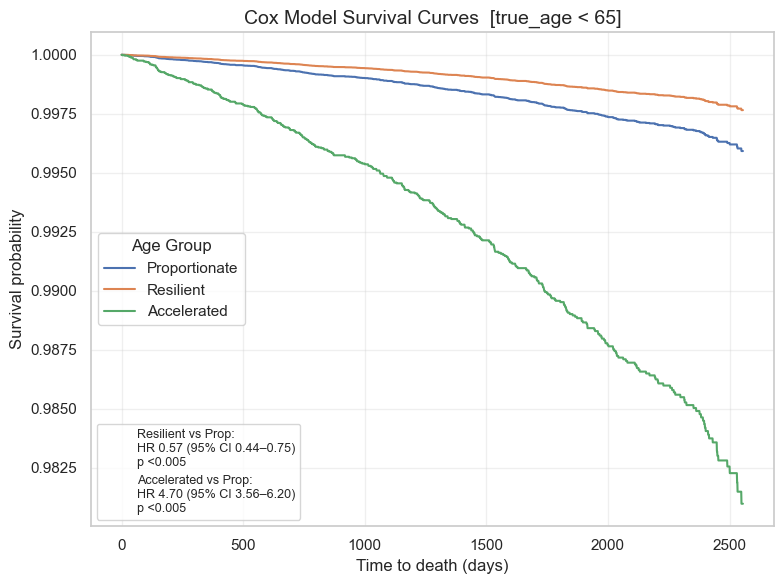

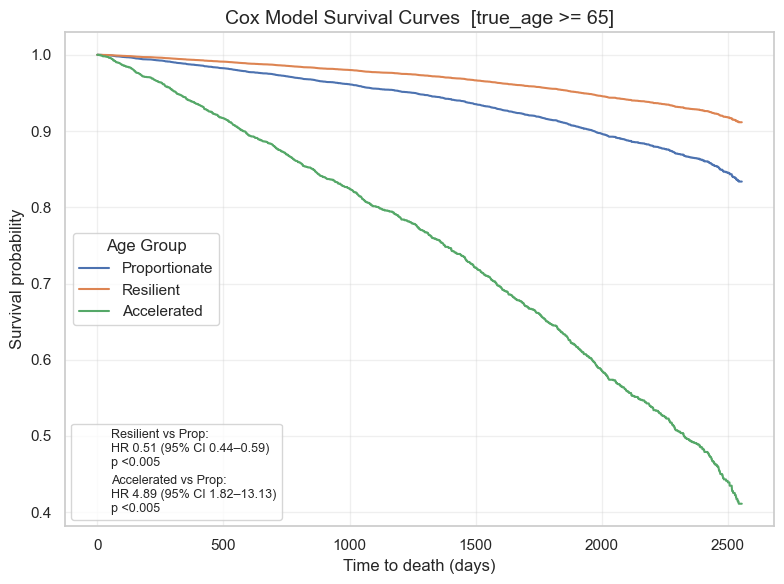


**Figure S3a and S3b:** Cox model survival curves stratified by chronological age group, adjusted for age, sex and, race and ethnicity. **S3a:** Patients younger than 65 years, **S3b:** Patients aged 65 years or older


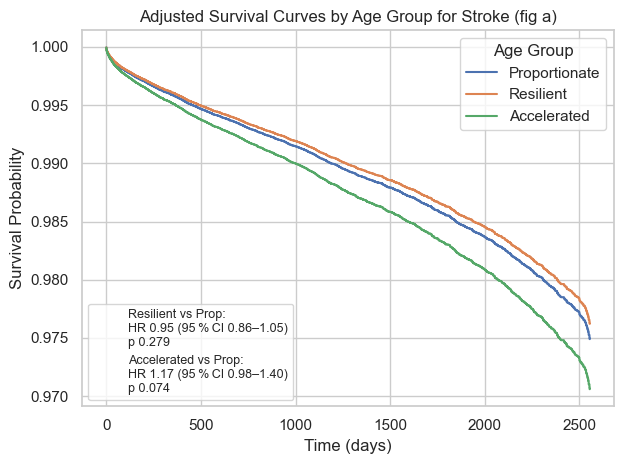

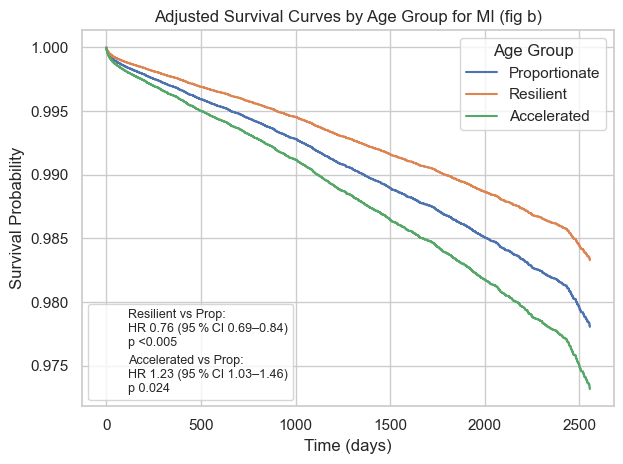

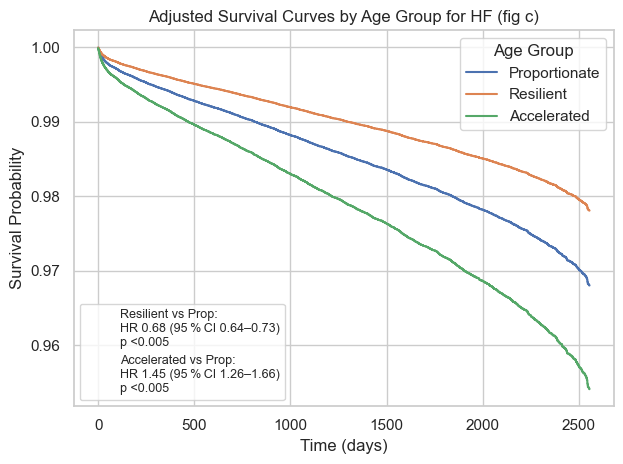


**Figure S4a–c:** Adjusted survival curves from Cox models for individual MACE outcomes, stratified by CBC delta age group. Models were adjusted for chronological age, sex, race and ethnicity, comorbidities, and social vulnerability index. **S4a:** Stroke, **S4b:** Myocardial infarction, **S4c:** Heart failure

**Table S1:** Incidence rates of MACE and mortality, stratified by CBC delta age group and true chronological age range

| **Age Group** | **Age Range** | **N** | **MACE Events** | **MACE PY** | **Death Events** | **Death PY** | **MACE Event Rate** | **Death Event Rate** |
| --- | --- | --- | --- | --- | --- | --- | --- | --- |
| **Proportionate** | <40 | 11,626 | 128 | 24,018.05 | 6 | 24,188.39 | 0.53 | 0.02 |
|  | 40–60 | 27,439 | 1,195 | 77,522.73 | 128 | 79,607.29 | 1.54 | 0.16 |
|  | 60–80 | 8,422 | 1,740 | 26,783.77 | 336 | 30,290.85 | 6.5 | 1.11 |
|  | >80 | 153 | 82 | 291.59 | 21 | 414.11 | 28.12 | 5.07 |
| **Resilient** | <40 | 12 | 0 | 31.61 | 0 | 31.61 | 0 | 0 |
|  | 40–60 | 6,113 | 302 | 15,771.69 | 30 | 16,298.18 | 1.91 | 0.18 |
|  | 60–80 | 20,646 | 3,246 | 58,735.27 | 475 | 64,730.39 | 5.53 | 0.73 |
|  | >80 | 4,099 | 1,615 | 9,369.40 | 395 | 11,907.24 | 17.24 | 3.32 |
| **Accelerated** | <40 | 26,867 | 234 | 59,531.29 | 29 | 59,871.70 | 0.39 | 0.05 |
|  | 40–60 | 4,022 | 290 | 12,116.50 | 60 | 12,672.10 | 2.39 | 0.47 |
|  | 60–80 | 118 | 39 | 357.53 | 16 | 427.44 | 10.91 | 3.74 |
|  | >80 | 0 | 0 | 0 | 0 | 0 | nan | nan |

**Table S2:** Subgroup analysis results from individual Cox models for each MACE component, showing hazard ratios for delta age groups (Resilient and Accelerated) compared to the Proportionate reference group. Models adjusted for true age, sex, race/ethnicity, comorbidities, and SVI.

| **Outcome** | **Variable** | **C-index** | **HR** | **CI lower 95%** | **CI upper 95%** | **P-value** |
| --- | --- | --- | --- | --- | --- | --- |
| **HF** | Resilient | 0.86 | 0.68 | 0.64 | 0.73 | <0.005 |
| **HF** | Accelerated | 0.86 | 1.45 | 1.26 | 1.66 | <0.005 |
| **MI** | Resilient | 0.83 | 0.76 | 0.69 | 0.84 | <0.005 |
| **MI** | Accelerated | 0.83 | 1.23 | 1.03 | 1.46 | 0.02 |
| **Stroke** | Resilient | 0.81 | 0.95 | 0.86 | 1.05 | 0.28 |
| **Stroke** | Accelerated | 0.81 | 1.17 | 0.98 | 1.4 | 0.07 |
| **Death** | Resilient | 0.86 | 0.59 | 0.52 | 0.68 | <0.005 |
| **Death** | Accelerated | 0.86 | 3.05 | 2.41 | 3.85 | <0.005 |

**Table S3:** Full subgroup analysis results from individual Cox models for each outcome included in MACE.

| **Outcome** | **C-index** | **Variable** | **HR (95% CI)** | **P-value** |
| --- | --- | --- | --- | --- |
| **HF** | 0.86 | True Age | 1.07 (1.07, 1.08) | <0.005 |
|  |  | Charlson Comorbidity Index | 1.58 (1.56, 1.61) | <0.005 |
|  |  | SVI (Social Vulnerability Index) | 1.00 (1.00, 1.00) | 0.56 |
|  |  | Resilient vs Proportionate | 0.68 (0.64, 0.73) | <0.005 |
|  |  | Accelerated vs Proportionate | 1.45 (1.26, 1.66) | <0.005 |
|  |  | Male (vs Female) | 1.36 (1.29, 1.44) | <0.005 |
|  |  | Non-Hispanic Asian (vs Hispanic) | 0.64 (0.53, 0.76) | <0.005 |
|  |  | Non-Hispanic Black (vs Hispanic) | 1.29 (1.17, 1.43) | <0.005 |
|  |  | Non-Hispanic Other (vs Hispanic) | 0.63 (0.51, 0.77) | <0.005 |
|  |  | Non-Hispanic White (vs Hispanic) | 0.85 (0.78, 0.93) | <0.005 |
| **MI** | 0.83 | True Age | 1.06 (1.06, 1.06) | <0.005 |
|  |  | Charlson Comorbidity Index | 1.51 (1.47, 1.54) | <0.005 |
|  |  | SVI (Social Vulnerability Index) | 1.00 (1.00, 1.00) | 0.98 |
|  |  | Resilient vs Proportionate | 0.76 (0.69, 0.84) | <0.005 |
|  |  | Accelerated vs Proportionate | 1.23 (1.03, 1.46) | 0.02 |
|  |  | Male (vs Female) | 1.65 (1.53, 1.77) | <0.005 |
|  |  | Non-Hispanic Asian (vs Hispanic) | 0.67 (0.53, 0.86) | <0.005 |
|  |  | Non-Hispanic Black (vs Hispanic) | 1.36 (1.18, 1.56) | <0.005 |
|  |  | Non-Hispanic Other (vs Hispanic) | 0.62 (0.47, 0.83) | <0.005 |
|  |  | Non-Hispanic White (vs Hispanic) | 0.99 (0.88, 1.11) | 0.85 |
| **Stroke** | 0.81 | True Age | 1.05 (1.05, 1.06) | <0.005 |
|  |  | Charlson Comorbidity Index | 1.48 (1.44, 1.51) | <0.005 |
|  |  | SVI (Social Vulnerability Index) | 1.00 (1.00, 1.00) | 0.35 |
|  |  | Resilient vs Proportionate | 0.95 (0.86, 1.05) | 0.28 |
|  |  | Accelerated vs Proportionate | 1.17 (0.98, 1.40) | 0.07 |
|  |  | Male (vs Female) | 1.12 (1.03, 1.21) | <0.005 |
|  |  | Non-Hispanic Asian (vs Hispanic) | 0.78 (0.62, 0.96) | 0.02 |
|  |  | Non-Hispanic Black (vs Hispanic) | 1.23 (1.08, 1.41) | <0.005 |
|  |  | Non-Hispanic Other (vs Hispanic) | 0.63 (0.48, 0.83) | <0.005 |
|  |  | Non-Hispanic White (vs Hispanic) | 0.84 (0.74, 0.94) | <0.005 |
| **Death** | 0.86 | True Age | 1.09 (1.09, 1.10) | <0.005 |
|  |  | Charlson Comorbidity Index | 1.45 (1.41, 1.50) | <0.005 |
|  |  | SVI (Social Vulnerability Index) | 1.00 (1.00, 1.00) | 0.76 |
|  |  | Resilient vs Proportionate | 0.59 (0.52, 0.68) | <0.005 |
|  |  | Accelerated vs Proportionate | 3.05 (2.41, 3.85) | <0.005 |
|  |  | Male (vs Female) | 1.47 (1.32, 1.62) | <0.005 |
|  |  | Non-Hispanic Asian (vs Hispanic) | 0.63 (0.45, 0.88) | 0.01 |
|  |  | Non-Hispanic Black (vs Hispanic) | 0.92 (0.75, 1.12) | 0.39 |
|  |  | Non-Hispanic Other (vs Hispanic) | 0.67 (0.46, 0.98) | 0.04 |
|  |  | Non-Hispanic White (vs Hispanic) | 0.84 (0.71, 0.98) | 0.03 |

**Table S4:** Combined Cox proportional hazard model results from three sequential Cox models for mortality, comparing two approaches to modeling HemeAge: categorical age group (Resilient and Accelerated vs. Proportionate) and continuous delta age.

| **Age Groups** | | | | | | |
| --- | --- | --- | --- | --- | --- | --- |
| **Model** | **C-index** | | **Variables** | **HR (95% CI)** | | **P-value** |
| **M1** | 0.84 | | True Age | 1.11 (1.11, 1.12) | | <0.005 |
|  | 0.84 | | Resilient vs Proportionate | 0.52 (0.45, 0.59) | | <0.005 |
|  |  | | Accelerated vs Proportionate | 3.60 (2.85, 4.55) | | <0.005 |
|  |  | | Male (vs Female) | 1.58 (1.42, 1.74) | | <0.005 |
|  |  | | Non-Hispanic Asian (vs Hispanic) | 0.57 (0.41, 0.80) | | <0.005 |
|  |  | | Non-Hispanic Black (vs Hispanic) | 1.05 (0.87, 1.28) | | 0.61 |
|  |  | | Non-Hispanic Other (vs Hispanic) | 0.62 (0.43, 0.91) | | 0.01 |
|  |  | | Non-Hispanic White (vs Hispanic) | 0.78 (0.66, 0.91) | | <0.005 |
| **M2** | 0.86 | | True Age | 1.09 (1.09, 1.10) | | <0.005 |
|  |  | | Charlson Comorbidity Index | 1.45 (1.41, 1.50) | | <0.005 |
|  |  | | Resilient vs Proportionate | 0.59 (0.52, 0.68) | | <0.005 |
|  |  | | Accelerated vs Proportionate | 3.05 (2.41, 3.85) | | <0.005 |
|  |  | | Male (vs Female) | 1.47 (1.32, 1.62) | | <0.005 |
|  |  | | Non-Hispanic Asian (vs Hispanic) | 0.63 (0.45, 0.88) | | 0.01 |
|  |  | | Non-Hispanic Black (vs Hispanic) | 0.92 (0.75, 1.12) | | 0.39 |
|  |  | | Non-Hispanic Other (vs Hispanic) | 0.67 (0.46, 0.98) | | 0.04 |
|  |  | | Non-Hispanic White (vs Hispanic) | 0.84 (0.71, 0.98) | | 0.03 |
| **M3** | 0.86 | | True Age | 1.09 (1.09, 1.10) | | <0.005 |
|  |  | | Charlson Comorbidity Index | 1.45 (1.41, 1.50) | | <0.005 |
|  |  | | SVI (Social Vulnerability Index) | 1.00 (1.00, 1.00) | | 0.76 |
|  |  | | Resilient vs Proportionate | 0.59 (0.52, 0.68) | | <0.005 |
|  |  | | Accelerated vs Proportionate | 3.05 (2.41, 3.85) | | <0.005 |
|  |  | | Male (vs Female) | 1.47 (1.32, 1.62) | | <0.005 |
|  |  | | Non-Hispanic Asian (vs Hispanic) | 0.63 (0.45, 0.88) | | 0.01 |
|  |  | | Non-Hispanic Black (vs Hispanic) | 0.92 (0.75, 1.12) | | 0.39 |
|  |  | | Non-Hispanic Other (vs Hispanic) | 0.67 (0.46, 0.98) | | 0.04 |
|  |  | | Non-Hispanic White (vs Hispanic) | 0.84 (0.71, 0.98) | | 0.03 |
| **Continuous Delta Age** | | | | | | |
| **M1** | | 0.84 | True Age | 1.13 (1.12, 1.13) | <0.005 | |
|  | |  | delta | 1.05 (1.04, 1.06) | <0.005 | |
|  | |  | Male (vs Female) | 1.43 (1.29, 1.59) | <0.005 | |
|  | |  | Non-Hispanic Asian (vs Hispanic) | 0.59 (0.42, 0.82) | <0.005 | |
|  | |  | Non-Hispanic Black (vs Hispanic) | 1.10 (0.90, 1.33) | 0.35 | |
|  | |  | Non-Hispanic Other (vs Hispanic) | 0.62 (0.42, 0.90) | 0.01 | |
|  | |  | Non-Hispanic White (vs Hispanic) | 0.74 (0.63, 0.87) | <0.005 | |
| **M2** | | 0.86 | True Age | 1.11 (1.10, 1.11) | <0.005 | |
|  | |  | delta | 1.04 (1.03, 1.05) | <0.005 | |
|  | |  | Charlson Comorbidity Index | 1.43 (1.39, 1.48) | <0.005 | |
|  | |  | Male (vs Female) | 1.37 (1.23, 1.52) | <0.005 | |
|  | |  | Non-Hispanic Asian (vs Hispanic) | 0.64 (0.46, 0.89) | 0.01 | |
|  | |  | Non-Hispanic Black (vs Hispanic) | 0.95 (0.78, 1.16) | 0.62 | |
|  | |  | Non-Hispanic Other (vs Hispanic) | 0.67 (0.46, 0.97) | 0.04 | |
|  | |  | Non-Hispanic White (vs Hispanic) | 0.81 (0.69, 0.95) | 0.01 | |
| **M3** | | 0.86 | True Age | 1.11 (1.10, 1.11) | <0.005 | |
|  | |  | delta | 1.04 (1.03, 1.05) | <0.005 | |
|  | |  | Charlson Comorbidity Index | 1.43 (1.39, 1.48) | <0.005 | |
|  | |  | SVI (Social Vulnerability Index) | 1.00 (1.00, 1.00) | 0.79 | |
|  | |  | Male (vs Female) | 1.37 (1.23, 1.52) | <0.005 | |
|  | |  | Non-Hispanic Asian (vs Hispanic) | 0.64 (0.46, 0.89) | 0.01 | |
|  | |  | Non-Hispanic Black (vs Hispanic) | 0.95 (0.78, 1.16) | 0.62 | |
|  | |  | Non-Hispanic Other (vs Hispanic) | 0.67 (0.46, 0.97) | 0.04 | |
|  | |  | Non-Hispanic White (vs Hispanic) | 0.81 (0.69, 0.95) | 0.01 | |

Model 1 (M1) adjusts for chronological age, sex, and race and ethnicity.
Model 2 (M2) adds comorbidities (Charlson comorbidity index).
Model 3 (M3) further includes social vulnerability index

**Table S5:** Combined Cox proportional hazard model results from three sequential Cox models for MACE, comparing two approaches to modeling biological aging: categorical age group and continuous delta age.

| **Age Groups** | | | | | |
| --- | --- | --- | --- | --- | --- |
| **Model** | **C-index** | **Variables** | **HR (95% CI)** | | **P-value** |
| **M1** | 0.80 | True Age | 1.08 (1.08, 1.09) | | <0.005 |
|  |  | Resilient vs Proportionate | 0.68 (0.64, 0.71) | | <0.005 |
|  |  | Accelerated vs Proportionate | 1.63 (1.48, 1.80) | | <0.005 |
|  |  | Male (vs Female) | 1.44 (1.38, 1.50) | | <0.005 |
|  |  | Non-Hispanic Asian (vs Hispanic) | 0.62 (0.55, 0.71) | | <0.005 |
|  |  | Non-Hispanic Black (vs Hispanic) | 1.42 (1.31, 1.53) | | <0.005 |
|  |  | Non-Hispanic Other (vs Hispanic) | 0.61 (0.52, 0.71) | | <0.005 |
|  |  | Non-Hispanic White (vs Hispanic) | 0.83 (0.77, 0.88) | | <0.005 |
| **M2** | 0.84 | True Age | 1.06 (1.06, 1.07) | | <0.005 |
|  |  | Charlson Comorbidity Index | 1.55 (1.53, 1.57) | | <0.005 |
|  |  | Resilient vs Proportionate | 0.76 (0.72, 0.81) | | <0.005 |
|  |  | Accelerated vs Proportionate | 1.37 (1.24, 1.51) | | <0.005 |
|  |  | Male (vs Female) | 1.35 (1.29, 1.41) | | <0.005 |
|  |  | Non-Hispanic Asian (vs Hispanic) | 0.68 (0.60, 0.78) | | <0.005 |
|  |  | Non-Hispanic Black (vs Hispanic) | 1.27 (1.18, 1.37) | | <0.005 |
|  |  | Non-Hispanic Other (vs Hispanic) | 0.66 (0.57, 0.76) | | <0.005 |
|  |  | Non-Hispanic White (vs Hispanic) | 0.89 (0.83, 0.95) | | <0.005 |
| **M3** | 0.84 | True Age | 1.06 (1.06, 1.07) | | <0.005 |
|  |  | Charlson Comorbidity Index | 1.55 (1.53, 1.57) | | <0.005 |
|  |  | SVI (Social Vulnerability Index) | 1.00 (1.00, 1.00) | | 0.1 |
|  |  | Resilient vs Proportionate | 0.76 (0.72, 0.81) | | <0.005 |
|  |  | Accelerated vs Proportionate | 1.37 (1.24, 1.51) | | <0.005 |
|  |  | Male (vs Female) | 1.35 (1.29, 1.41) | | <0.005 |
|  |  | Non-Hispanic Asian (vs Hispanic) | 0.68 (0.60, 0.78) | | <0.005 |
|  |  | Non-Hispanic Black (vs Hispanic) | 1.27 (1.18, 1.37) | | <0.005 |
|  |  | Non-Hispanic Other (vs Hispanic) | 0.66 (0.57, 0.76) | | <0.005 |
|  |  | Non-Hispanic White (vs Hispanic) | 0.89 (0.83, 0.95) | | <0.005 |
| **Continuous Delta Age** | | | | | |
| **M1** | 0.8 | True Age | | 1.09 (1.09, 1.10) | <0.005 |
|  |  | Delta Age | | 1.03 (1.03, 1.03) | <0.005 |
|  |  | Male (vs Female) | | 1.38 (1.32, 1.44) | <0.005 |
|  |  | Non-Hispanic Asian (vs Hispanic) | | 0.63 (0.55, 0.72) | <0.005 |
|  |  | Non-Hispanic Black (vs Hispanic) | | 1.45 (1.34, 1.57) | <0.005 |
|  |  | Non-Hispanic Other (vs Hispanic) | | 0.61 (0.52, 0.71) | <0.005 |
|  |  | Non-Hispanic White (vs Hispanic) | | 0.81 (0.75, 0.86) | <0.005 |
| **M2** | 0.84 | True Age | | 1.07 (1.07, 1.07) | <0.005 |
|  |  | Delta Age | | 1.02 (1.02, 1.02) | <0.005 |
|  |  | Charlson Comorbidity Index | | 1.54 (1.52, 1.56) | <0.005 |
|  |  | Male (vs Female) | | 1.32 (1.26, 1.37) | <0.005 |
|  |  | Non-Hispanic Asian (vs Hispanic) | | 0.69 (0.60, 0.78) | <0.005 |
|  |  | Non-Hispanic Black (vs Hispanic) | | 1.29 (1.20, 1.40) | <0.005 |
|  |  | Non-Hispanic Other (vs Hispanic) | | 0.66 (0.57, 0.77) | <0.005 |
|  |  | Non-Hispanic White (vs Hispanic) | | 0.88 (0.82, 0.94) | <0.005 |
| **M3** | 0.84 | True Age | | 1.07 (1.07, 1.07) | <0.005 |
|  |  | Delta Age | | 1.02 (1.02, 1.02) | <0.005 |
|  |  | Charlson Comorbidity Index | | 1.54 (1.52, 1.56) | <0.005 |
|  |  | SVI (Social Vulnerability Index) | | 1.00 (1.00, 1.00) | 0.1 |
|  |  | Male (vs Female) | | 1.32 (1.26, 1.37) | <0.005 |
|  |  | Non-Hispanic Asian (vs Hispanic) | | 0.69 (0.60, 0.78) | <0.005 |
|  |  | Non-Hispanic Black (vs Hispanic) | | 1.29 (1.20, 1.40) | <0.005 |
|  |  | Non-Hispanic Other (vs Hispanic) | | 0.66 (0.57, 0.77) | <0.005 |
|  |  | Non-Hispanic White (vs Hispanic) | | 0.88 (0.82, 0.94) | <0.005 |

Model 1 (M1) adjusts for chronological age, sex, and race and ethnicity.
Model 2 (M2) includes M1 covariates plus comorbidities (Charlson comorbidity index).
Model 3 (M3) further includes social vulnerability index.

| **Age (Years)** | **Accelerated** | | | **Resilient** | | |
| --- | --- | --- | --- | --- | --- | --- |
|  | **HR** | **95% CI** | **P Value** | **HR** | **95% CI** | **P Value** |
| **Mortality** | | | | | | |
| <40 | 104.82 | (1.66, 6614.35) | 0.028 |  |  |  |
| 40-60 | 3.03 | (2.17, 4.22) | <0.005 | 0.83 | (0.55, 1.25) | 0.383 |
| 60-80 | 3.61 | (2.17, 6.00) | <0.005 | 0.59 | (0.50, 0.68) | <0.005 |
| >80 |  |  |  | 0.59 | (0.37, 0.92) | 0.02 |
| **MACE** | | | | | | |
| <40 | 1.07 | (0.85, 1.36) | 0.565 |  |  |  |
| 40-60 | 1.61 | (1.40, 1.84) | <0.005 | 0.89 | (0.78, 1.02) | 0.099 |
| 60-80 | 1.53 | (1.11, 2.10) | 0.009 | 0.77 | (0.73, 0.82) | <0.005 |
| >80 |  |  |  | 0.55 | (0.44, 0.69) | <0.005 |

**Table S6.** Age-stratified Cox proportional hazards models for mortality and major adverse cardiovascular events additionally adjusted for Charlson Comorbidity Index (CCI), age, sex, and race/ethnicity.
